# Complete remission of locoregionally metastatic melanoma after one single dose of pembrolizumab: A case report

**DOI:** 10.1016/j.jdcr.2025.01.035

**Published:** 2025-03-04

**Authors:** Dong Ren, Thuy B. Tran, Warren A. Chow, Shelby Barnachea, Katherine Wei, Bonnie A. Lee

**Affiliations:** a Department of Pathology and Laboratory Medicine, University of California, Irvine, Irvine, California; b Division of Surgical Oncology, University of California, Irvine, Irvine, California; c Division of Hematology/Oncology, University of California, Irvine, Irvine, California; d Department of Radiology, University of California, Irvine, Irvine, California; e Departments of Dermatology and Pathology, University of California, Irvine, Irvine, California

**Keywords:** complete remission, locoregionally metastatic melanoma, pembrolizumab, single dose

## Introduction

Despite comprising just 1% of skin malignancies, melanoma is responsible for roughly 90% of skin cancer-related deaths because of its high tendency to metastasize and its aggressive clinical behavior.^1^ Patients with metastatic melanoma had a dismal prognosis with a 5-year survival rate of <5% and a median survival of only 6 to 9 months before the introduction of immunotherapy.^2^ Several immunotherapeutic drugs, including pembrolizumab (Keytruda) and nivolumab (Opdivo) were approved by the United States Food and Drug Administration in 2014 for treating metastatic melanoma. These treatments have significantly enhanced overall survival, with 5-year survival rates of 34% to 41% and median overall survival ranging from 22.6 to 38.6 months.^3^^,^^4^

The standard of care for metastatic melanoma includes a protocol-specified course of pembrolizumab every 3 weeks for up to 2 years. Data from the KEYNOTE-006 trial showed that the majority of 834 patients with metastatic melanoma (74%) who completed 2 years of pembrolizumab treatment demonstrated variable responses, including complete response (20%), partial response (67%), and stable disease (13%).^5^ Interestingly, Jansen et al^6^ published a cohort analysis of patients who discontinued pembrolizumab before 2 years of therapy that showed 63% of 185 patients with metastatic melanoma achieved complete regression, with 24% showing a partial response and 9% having stable disease after discontinuation of therapy.

There is data to suggest that desmoplastic type melanomas respond well to therapy. Notably, Sondak et al^7^ recently presented data that patients with nonmetastatic desmoplastic melanoma exhibited pathologic complete response to neoadjuvant pembrolizumab at an impressive 58% (16/28) rate, including one patient who achieved a complete response after receiving only a single dose, with therapy limited by immunotherapy-related colitis. Additionally, Kendra et al^8^ reported that 9 of 27 patients (33%) with unresectable metastatic desmoplastic melanoma achieved a complete response to pembrolizumab (*P* < .001) after a median of 15 cycles.^8^ Here, we present the first case, to our knowledge, of locoregionally metastatic mixed spindle cell and desmoplastic melanoma involving the right supraclavicular lymph node, achieving a complete response after a single dose of pembrolizumab, with no disease progression identified at 1-year follow-up.

## Case report

**Fig 1:**
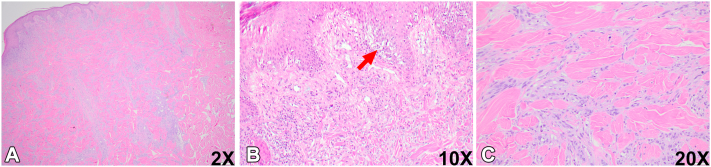
Histologic evaluation of the primary melanoma lesion on the mid-upper back. **A-C,** Representative section of the primary melanoma lesion. *Red arrows* in (**B**) indicate the melanoma in situ component. *SUV*, Standardized uptake value.

**Fig 2:**
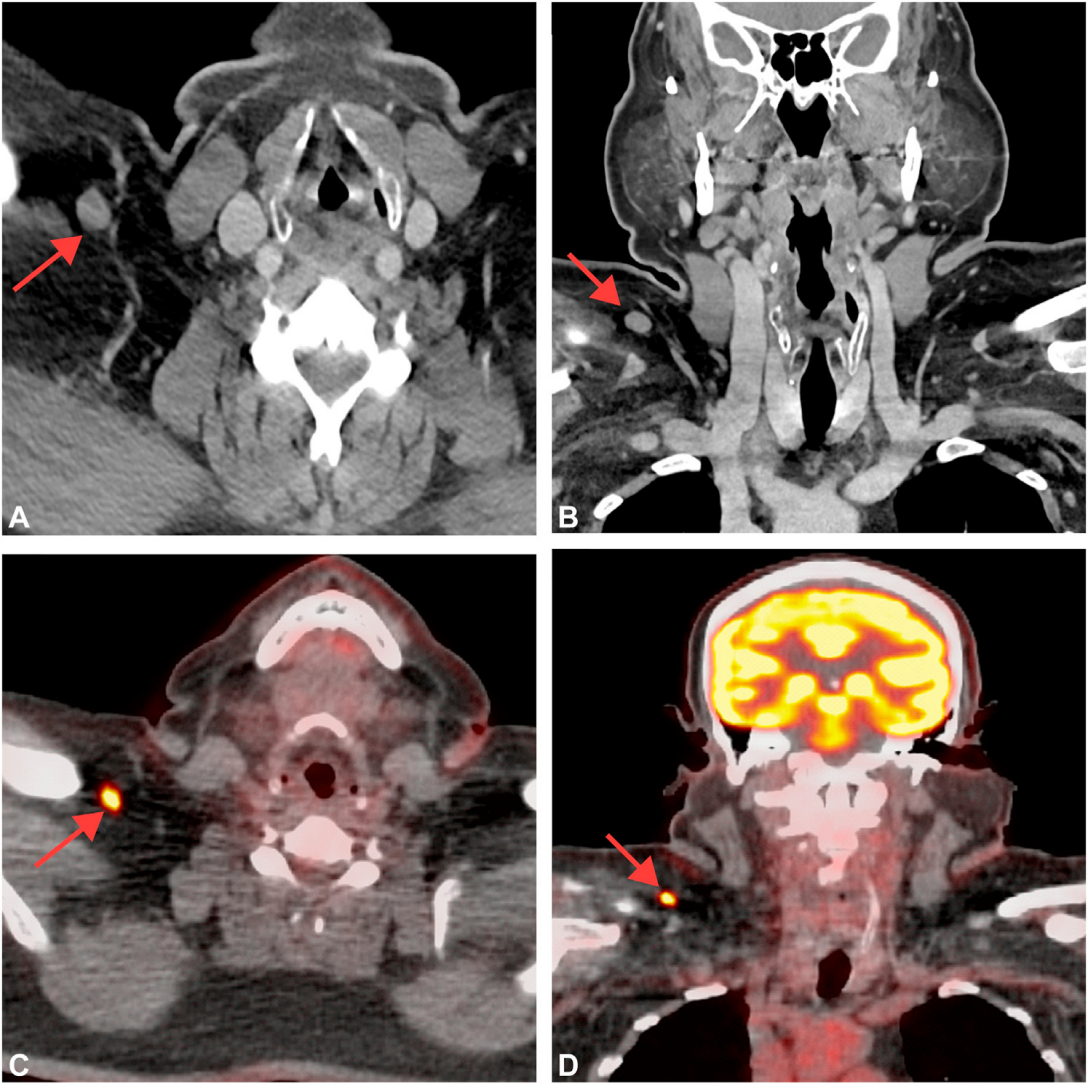
Immediate imaging evaluation of the neck after the biopsy. **A** and **B,** Axial (**A**) and coronal (**B**) images from the patient's computed tomography (CT) neck with contrast demonstrates an enlarged, approximately 13 mm short axis right supraclavicular lymph node (*red arrows*), suspicious for nodal metastatic disease; **C** and **D,** Axial (**C**) and coronal (**D**) images from the patient's PET-CT scan demonstrates F-18 fluorodeoxyglucose (FDG) avidity of the right supraclavicular lymph node with maximal FDG activity measuring 7 Standardized Uptake Value (SUV) (*red arrows*), suggestive of nodal metastatic disease.

A 60-year-old man with history of melanoma on the upper portion of the left arm diagnosed 9 years prior presented to our institution for treatment of a new melanoma on the mid-upper back, which was a nonulcerated mixed spindled and desmoplastic melanoma (10.1 mm, pT4a) (Fig 1, *A-C*). The mitotic rate was >15 mitoses per mm^2^ and nonbrisk tumor-infiltrating lymphocytes were present. The tumor cells expressed Sox-10 and S100, but were negative for Melan-A. Computed tomography (CT) imaging of the neck at 1 week showed a slightly enlarged right supraclavicular lymph node measuring 13 mm, suspicious for metastatic melanoma (Fig 2, *A* and *B*). CT imaging of the chest and abdomen revealed several lucent bone lesions in the thoracic vertebral bodies. There was no intracranial metastatic disease identified on brain magnetic resonance imaging. Subsequent positron emission tomography-CT scan showed an F-18 fluorodeoxyglucose (FDG) avid right supraclavicular lymph node, suspicious for metastatic disease, and no additional FDG avid lesions were identified (Fig 2, *C* and *D*).

**Fig 3:**
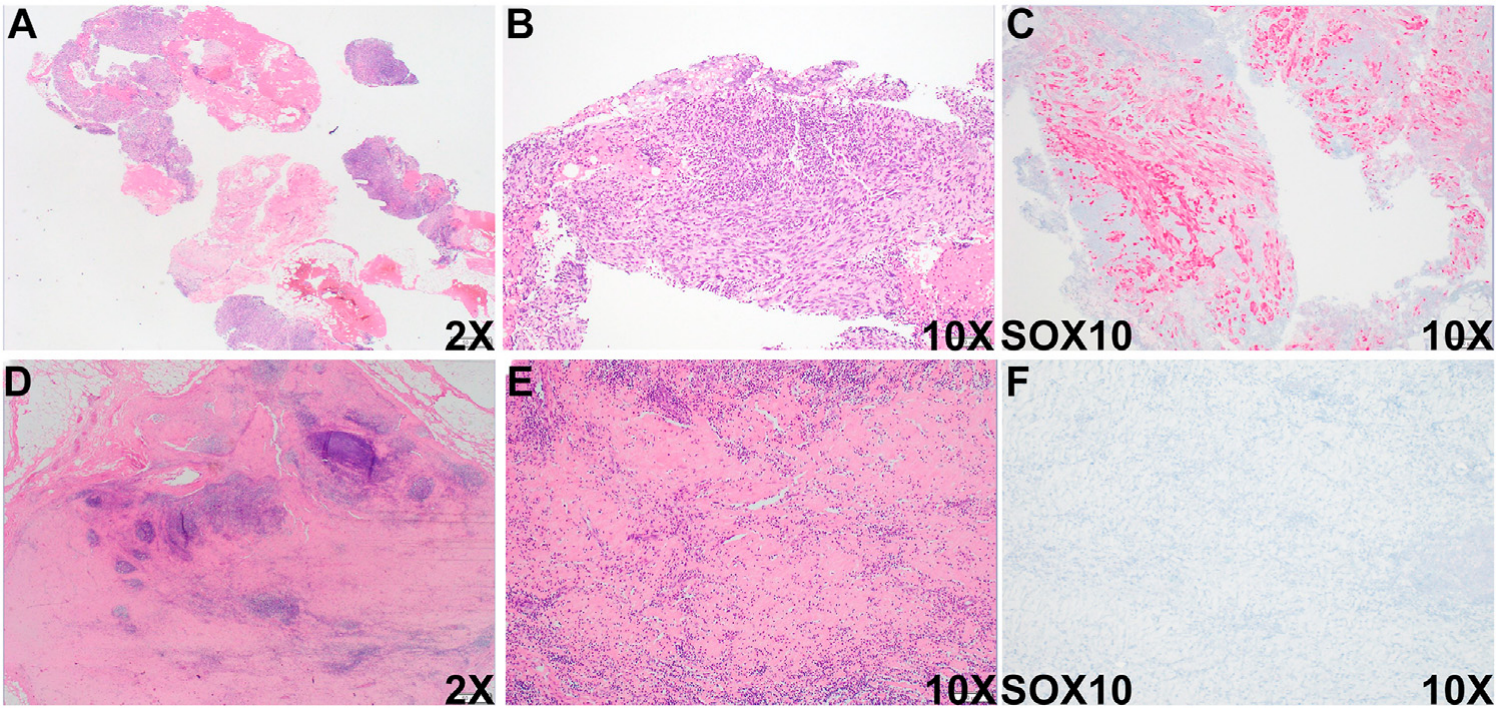
Histologic evaluation of the right supraclavicular lymph node before and after pembrolizumab treatment. Representative sections of the right supraclavicular lymph node and immunohistochemical staining with SOX10 before (**A-C**) and after (**D-F**) treatment.

**Fig 4:**
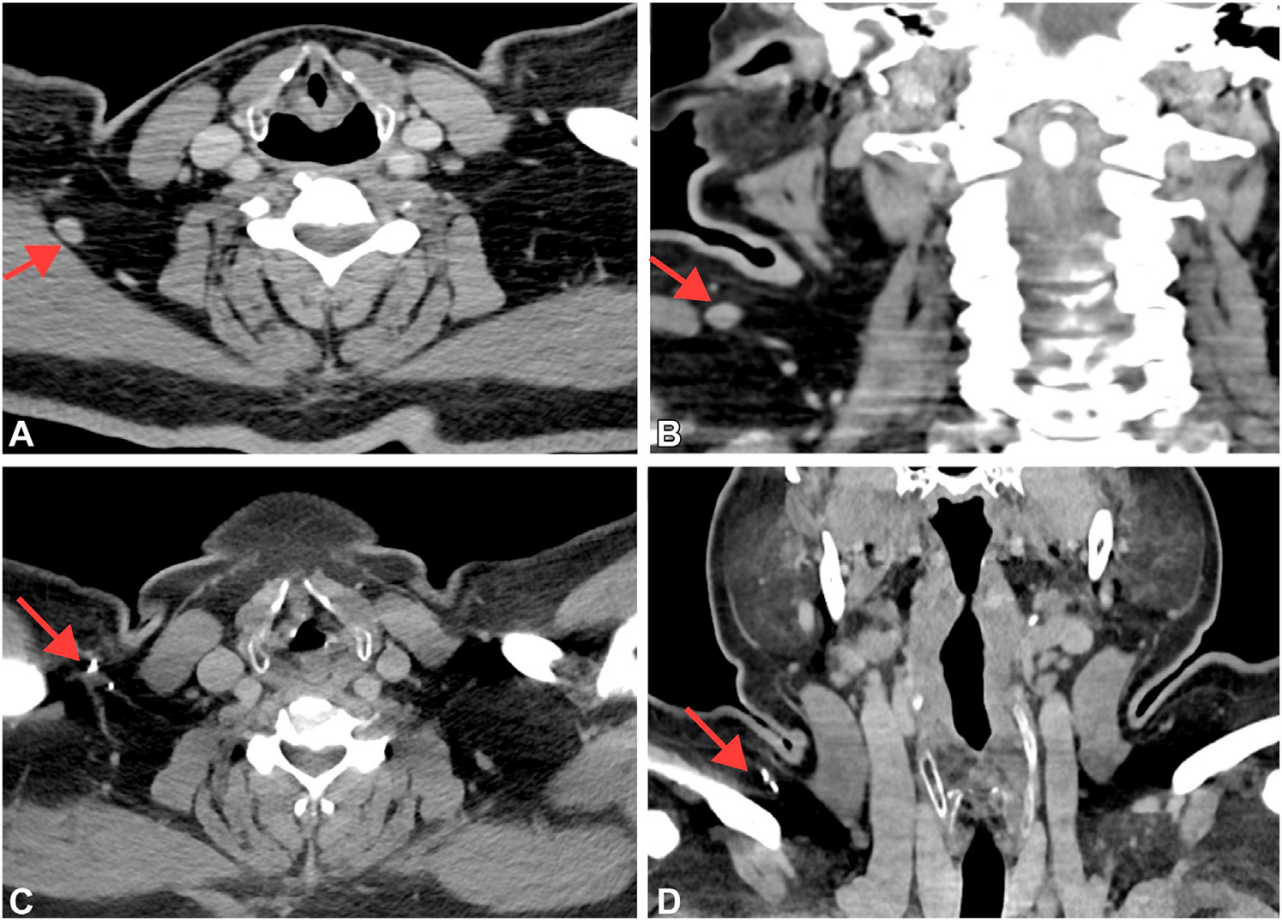
Radiologic evaluation of the lesion before and after treatment. Axial (**A**) and coronal (**B**) images after one month of pembrolizumab treatment demonstrates a new prominent 8 mm short axis right supraclavicular lymph node slightly posteroinferior to the original 13 mm right supraclavicular lymph node (*red arrows*), suggesting additional nodal metastases. Axial (**C**) and coronal (**D**) images of the neck from the patient's contrast-enhanced computed tomography (CT) one year later post Pembrolizumab treatment continues to show complete remission without evidence of disease recurrence or metastatic disease. Contrast-enhanced CT of the chest, abdomen, and pelvis (not shown) also similarly showed no evidence for metastatic disease.

Biopsy of the right supraclavicular lymph node demonstrated moderately pleomorphic oval to spindled cells arranged in sheets and fascicles within a lymphoid aggregate (Fig 3, *A* and *B*). Immunohistochemistry revealed diffuse and strong positivity for SOX10 in tumor cells, consistent with metastatic spindle cell melanoma (Fig 3*C*). The patient initiated one dose of pembrolizumab but had to discontinue due to immunotherapy-related pneumonitis. Histologic examination of the right supraclavicular lymph node one-year post-treatment revealed a sclerotic node with multiple foci of lymphoid aggregates interspersed throughout the lymph node tissue (Fig 3, *D* and *E*), and SOX10 staining failed to demonstrate evidence of melanoma (Fig 3*F*). Consistently, follow-up CT imaging of the neck one-month later showed an additional 8 mm right supraclavicular lymph node alongside the stable 13 mm right supraclavicular lymph node (Fig 4, *A* and *B*). However, follow-up CT imaging of the neck, chest, and abdomen one-year post-treatment demonstrated that the patient remains in complete remission, with no definitive evidence of metastatic disease (Fig 4, *C* and *D*). These radiological and pathological findings collectively suggest a complete response of locoregionally metastatic melanoma to pembrolizumab treatment.

## Discussion

Metastatic melanoma patients with a complete response to pembrolizumab treatment typically experience favorable overall survival. Data from the KEYNOTE-006 trial indicated that patients with advanced melanoma who achieved a complete response to therapy had a 100% 3-year overall survival rate.^5^ Our patient exhibited both radiologic and pathologic complete response after just 1 cycle of pembrolizumab, and has showed no signs of suspicious tumor progression in the 1-year follow-up. In the study by Jansen et al,^6^ only 14% of patients with a complete response at the time of treatment discontinuation experienced tumor progression, compared with 32% with a partial response and 50% with stable disease. These findings shed a positive light on our patient’s likely prognosis.

Tertiary lymphoid structures characterized by distinct clusters of B cells, CD8+ T cells, CD83+ dendritic cells in T-cell zones, and PNAd-positive vasculature resembling high endothelial venules, have been variably reported in desmoplastic melanoma and are associated with predicting tumor response to immunotherapy.^9^ In this case, we did not identify lymphoid aggregates within the mixed spindled and desmoplastic melanoma component, although non-brisk tumor-infiltrating lymphocytes were present.

The unrestricted cytotoxic activity due to the loss of T cell inhibition imposed by immunotherapy can lead to various immune-related side effects known as immune-related adverse events.^10^ The most commonly affected sites for pembrolizumab-related immune-related adverse events include the skin, gastrointestinal tract, hepatic tract, and endocrine system,^3^^,^^10^ with affected patients typically experiencing rash, pruritus, diarrhea, and endocrine abnormalities.^11^ Several studies have reported that discontinuation of immunotherapy because of immune-related adverse events is the main cause of tumor recurrence and progression in patients with melanoma.^4^^,^^6^ Our patient experienced a relatively uncommon adverse event of pneumonitis related to pembrolizumab treatment and had to stop therapy after a single dose.

To our knowledge, this is the first report of a patient with locoregionally metastatic melanoma experiencing a complete response after a single cycle of pembrolizumab, supporting data demonstrating increased efficacy among patients with a desmoplastic subtype and further highlighting the pivotal efficacy of pembrolizumab in the treatment of advanced disease.

## Declaration of generative AI and AI-assisted technologies in the writing process

During the preparation of this work the author(s) used ChatGPT in order to check for typos and grammatical issues only. After using this tool/service, the author(s) reviewed and edited the content as needed and take(s) full responsibility for the content of the publication.

## Conflicts of interest

None disclosed.
